# Optic Nerve Head and Retinal Nerve Fiber Layer Analysis in Emmetropic Malay Children

**DOI:** 10.7759/cureus.53890

**Published:** 2024-02-09

**Authors:** Ahmad Amelia, Ismail Fatimah-Halwani, Ismail Shatriah

**Affiliations:** 1 Department of Ophthalmology, Hospital Sultan Ismail Petra, Kuala Krai, MYS; 2 Department of Ophthalmology and Visual Science, School of Medical Sciences, Universiti Sains Malaysia, Kota Bharu, MYS; 3 Ophthalmology Clinic, Hospital Universiti Sains Malaysia, Kota Bharu, MYS; 4 Department of Ophthalmology and Visual Science, School of Medical Sciences, Universiti Sains Malaysia, Kubang Kerian, MYS

**Keywords:** (rnflt) thickness, retinal nerve fiber layer, optic nerve head parameters, malay children, emmetropic

## Abstract

Background and objectives: The study aims to investigate the normal reference values for optic nerve head (ONH) and retinal nerve fiber layer (RNFL) parameters in emmetropic Malay children, utilizing measurements obtained through Cirrus SD-OCT (Carl Zeiss Meditec, Dublin, CA).

Methodology: A cross-sectional study was conducted on 95 Malay children, aged between seven and 17 years, with no ocular abnormalities. It was held at Hospital Universiti Sains Malaysia, Malaysia, from January 2014 to December 2015. All children underwent a full ocular examination, including refraction and calculation of axial length. ONH and retinal nerve fiber layer thickness (RNFLT) parameters were measured using the Cirrus SD-OCT machine. One eye of each subject was selected randomly for study. The associations between the parameters and the effect of age, gender, axial length, and spherical equivalent (SE) on the measurements were statistically validated.

Results: Ninety-five children were involved in the study, with 65 females (68.4%) and 30 males (31.6%). The mean age was 10.6 (2.82) years, the mean intraocular pressure (IOP) was 14.8 (2.81) mmHg, the mean SE-refraction was 0.12 (0.28) diopters, and the mean axial length was 23.03 (0.76) mm. The mean disc area, rim area, and cup volume were 2.32 (0.40) mm^2^, 1.53 (0.33) mm^2^, and 0.204 (0.16) mm^3^, respectively. The average cup-to-disc ratio (SD) (CDR) and the vertical CDR were 0.55 (0.13) and 0.50 (0.14). Mean (SD) RNFLT was 102.08 (11.08) μm for all patients. There was a strong positive correlation between the average, superior, and inferior RNFLT with the optical disc area. The rim area and the average, superior, inferior, and nasal RNFLT also showed a significant correlation. The inferior RNFLT was negatively correlated with the average CDR. There was also a major influence of gender on the disc area. There were no major age, axial length, and SE influences on the measurements.

Conclusions: This study provided normative information for ONH and RNFLT parameters in emmetropic Malay children. It was observed that emmetropic Malay males exhibited a significantly larger optical disc area. The increase in RNFLT is correlated with a significant increase in disc and rim areas.

## Introduction

Numerous studies have been performed in the past to ascertain the normative value for optic nerve head (ONH) and retinal nerve fiber layer thickness (RNFLT) parameters among childhood populations worldwide, using various modalities [1-10]. However, the bulk of these findings are limited to the East Asian and Middle Eastern communities [2,4,5,7,10]. Just one study in the Southeast Asian zone analyzed these data using a Heidelberg Retinal Tomography (HRT) machine, primarily involving Chinese Singaporean children [11].

The Malay ethnic community constitutes a large proportion of the population in Southeast Asia, with about 300 to 400 million population [12]. However, none of the data recorded normative values for ONH and RNFLT in the Malay community except for one adult study mentioned in Singapore [13].

The normative data for children using Cirrus SD-OCT (Carl Zeiss Meditec, Dublin, CA) are less documented, particularly about ONH parameters. Previous studies have reported a correlation between RNFLT and ONH parameters using OCT [4]. This is important for a better evaluation of the ONH and RNFLT parameters, as several authors have identified a substantial increase in RNFLT with an increase in disc areas [4].

The goal of this study was to report normative values for ONH and RNFLT parameters in emmetropic Malay children and analyze associations between these parameters using Cirrus SD-OCT. The effect of age, gender, axial length, and refraction on these parameters has also been analyzed as additional insight into future studies. As of now, this study marks the first attempt to identify normative values and correlations of ONH and RNFLT parameters using Cirrus SD-OCT in children in Southeast Asia.

## Materials and methods

We conducted a hospital-based, cross-sectional study involving 95 emmetropic Malay children, aged between 7 and 17 years, who attended the Eye Clinic at Hospital Universiti Sains Malaysia from January 2014 to December 2015. This study protocol was accepted by the Research and Ethics Committee (USMKK/PPP/JEPeM (223.4[2.7]), the School of Medical Sciences, Hospital Universiti Sains Malaysia, and all procedures were conducted by the Helsinki Declaration. Documented written consent was acquired from both parents or guardians before the enrollment of subjects.

We enlisted Malay children who met the following inclusion criteria: Malay children aged between seven and 17 without ocular abnormalities, best-corrected visual acuity (BCVA) better than 6/12 (20/40), intraocular pressure (IOP) equal to or less than 21 mmHg, with the spherical equivalent (SE) between ±0.5 DS (Diopter Sphere) (emmetropia), and astigmatism less than -1.00 DC. Exclusion criteria included subjects with a history of optic nerve anomalies or retinal diseases, a history of glaucoma (primary, secondary, or family history), a history of intraocular surgery, prematurity, ocular trauma, a history of neurological disease, syndromic children, and children with strabismus or amblyopia.

All children went through a complete ocular examination, including distant visual acuity with a Snellen chart, ocular motility, and examination of the external eye structure, cornea, and anterior chamber using a slit-lamp. IOP was measured for both eyes using a Goldman application tonometer or air puff tonometer (Reichert, NY, USA). Then the axial length measurement was completed by the IOL Master (Carl Zeiss Meditec, Jena, Germany). Topical cyclopentolate 1% was instilled into both eyes three times, with a five-minute interval, before refractive assessment. Cycloplegic refraction was done by a trained optometrist using an autorefractor (Reichert, NY), and dilated fundus examinations were performed.

Cirrus SD-OCT was used in this study for ONH parameters quantitative assessment, including disc area, rim area, cup volume, average cup-to-disc ratio (CDR), vertical CDR, and RNFL measurement, including average RNFL, inferior quadrant, superior quadrant, nasal quadrant, and temporal quadrant. It has a 5 µm axial resolution and scans at 27,000 A-scans per second. The measurements were obtained using the Optic Disc Cube 200 x 200 protocol. A diameter calculation circle of 3.46 mm around the center of the optic disc was automatically placed using a Cirrus software algorithm. The entire case used the internal fixation target. The scan was centrally placed on the optic disc and was verified by direct observation of fundus structures on a video screen by a single trained co-investigator.

Satisfactory image quality criteria comprised images with optimal centration of the optic disc, absence of blinking or eye movement artifacts, and a signal strength ≥7. Inadequate quality and poorly focused images were not accepted, and the procedure was repeated until acceptable images were obtained. If, even after five attempts, the image is still unsatisfactory, the procedure is abandoned and repeated at another time. The selection of one eye of each subject was done randomly for analysis.

The demographic data, clinical findings ONH parameters, and RNFLT measurement details were collected in a separate data collection sheet. Data entry and analysis were done by using IBM SPSS Statistics for Windows, Version 22.0 (IBM Corp., Armonk, NY). Means and standard deviations (SD) were reported for normally distributed data, while medians and interquartile ranges were defined for non-normally distributed data. The correlation between the ONH parameters and the RNFLT was rendered using the Pearson correlation coefficient.

An independent t-test was carried out to compare measurements between different genders. Univariate and multivariate regression analyses were performed to determine the potential impact of age, gender, axial length, and SE on measurements. The *P*-value of less than 0.05 was found to be statistically significant.

## Results

We finally enrolled a total of 95 subjects who fulfilled the inclusion criteria in the study. Table 1 shows the demographic data and clinical characteristics of the study subjects. The age (mean [SD]) of subjects was 10.6 (2.82) years. Of the total participants, 30 were boys (31.6%) and 65 were girls (68.4%). Among them, 71 subjects (74.7%) exhibited a visual acuity of 6/6 (20/20), while the remaining 24 subjects (25.3%) had a visual acuity of 6/7.5 (20/25). The mean IOP was 14.8 (±2.81) mmHg with a range of 10-20 mmHg. The mean SE refraction was -0.12 (±0.28) diopters, ranging between -0.50 and +0.50 diopters. The average axial length was 23.03 (±0.76) mm with a range of 21.37 to 24.90 mm.

**Table 1:** Demographic and clinical characteristics (n = 95). ^*^n (%). BCVA, best-corrected visual acuity; SD, standard deviation

Characteristics	Children with emmetropia, mean (SD)
Mean age (years)	10.6 (2.82)
7-10	50 (52.6)*
11-17	45 (47.4)*
Gender (%)	
Male	30 (31.6)*
Female	65 (68.4)*
BCVA (%)	
6/6 (20/20)	71 (74.7)*
6/7.5 (20/25)	24 (25.6)*
6/9 (or worse)	0 (0.0)*
Mean intraocular pressure (mmHg)	
Range 10-20	14.8 (2.81)
Mean spherical equivalent (diopter)	
Range -0.50 to +0.50	-0.12 (0.28)
Mean axial length (mm)	
Range 21.37-24.90	23.03 (0.76)

Subjects were subdivided into two subgroups based on age. The first group comprises subjects aged seven to 10 years (*n *= 50), while the second group consists of subjects aged 11 to 17 years (*n* = 45), facilitating a clearer presentation of the data. Table 2 shows the distribution of ONH parameters and RNFLT measurements according to age groups. The disc area (mean [SD]), rim area (mean [SD]), and cup volume (mean [SD]) for all subjects were 2.32 (0.40) mm^2^, 1.53 (0.33) mm^2^, and 0.204 (0.16) mm^3^, respectively. The average CDR (SD) and vertical CDR were 0.55 (0.13) and 0.50 (0.14), respectively. RNFLT (mean [SD]) for all subjects was 102.08 (11.08) µm. The thickest region was the inferior RNFL, followed by the superior and temporal regions, with the nasal RNFL being the thinnest.

**Table 2:** Distribution of optic nerve head parameters and retinal nerve fiber layer thickness (n = 95). DA, disc area; RA, rim area; CV, cup volume; CDR, cup-to-disc ratio; ONH, optic nerve head; SD, standard deviation

Age group (years)	All ages (*n *= 95), mean (SD)	7-10 years (*n *= 50), mean (SD)	11-17 years (*n *= 45), mean (SD)
ONH parameters			
Disc area (mm^2^)	2.32 (0.40)	2.35 (0.41)	2.28 (0.39)
Rim area (mm^2^)	1.53 (0.33)	1.56 (0.34)	1.50 (0.32)
Cup volume (mm^3^)	0.204 (0.16)	0.190 (0.14)	0.219 (0.17)
Average cup-to-disc ratio	0.55 (0.13)	0.55 (0.14)	0.56 (0.13)
Vertical cup-to-disc ratio	0.50 (0.14)	0.50 (0.14)	0.50 (0.14)
RNFL (µm)			
Average	102.08 (11.08)	101.92 (11.49)	102.27 (10.73)
Superior	131.05 (20.02)	131.20 (19.89)	130.89 (20.38)
Temporal	72.24 (11.06)	73.40 (12.28)	70.95 (9.50)
Inferior	134.14 (18.54)	133.38 (21.33)	134.98 (15.06)
Nasal	70.71 (12.36)	69.48 (11.42)	72.07 (13.32)

Table 3 shows a correlation between ONH and RNFLT parameters. The average (*r* = 0.363, *P* = 0.000), superior (*r *= 0.304, *P *= 0.003), and inferior (*r *= 0.299, *P *= 0.003) RNFLT showed a strong positive correlation with the optic disc area. A significant correlation was observed between the rim area and the average, superior, inferior, and nasal RNFLT (*r *= 0.445, *P *= 0.000), (*r *= 0.306, *P *= 0.003), (*r *= 0.409, *P *= 0.000), (*r *= 0.305, *P *= 0.003), respectively.

**Table 3:** Correlation (r) between optic nerve head parameters and retinal nerve fiber layer thickness (n = 95). ^*^Correlation is significant at the 0.05 level (two-tailed). CDR, cup-to-disc ratio; RNFL, retinal nerve fiber layer

	Disc area	Rim area	Cup volume	Vertical CDR	Average RNFL	Superior RNFL	Temporal RNFL	Inferior RNFL	Nasal RNFL
Average CDR	0.302*	-0.656*	0.790*	0.945*	-0.188	-0.083	-0.024	-0.238*	-0.147
Disc area		0.473*	0.446*	0.328*	0.363*	0.304*	0.176	0.299*	0.195
Rim area			-0.488*	-0.578*	0.445*	0.306*	0.186	0.409*	0.305*
Cup volume				0.729*	-0.062	-0.027	-0.054	-0.029	-0.089
Vertical CDR					-0.146	-0.041	-0.062	-0.175	-0.125
Average RNFL						0.820*	0.387*	0.773*	0.739*
Superior RNFL							0.174	0.416*	0.517*
Temporal RNFL								0.103	0.074
Inferior RNFL									0.507*

We observed a significant gender effect on the disc area (*b *= 0.281, *P *= 0.0010) (Table 4). Male gender revealed a larger disc area as compared to females. No significant correlations were observed between all other variables on ONH and RNFLT measurements.

**Table 4:** Factors affecting the disc area in emmetropic Malay children (n = 95). ^*^P < 0.05 indicates a significant association with the disc area.

Variables	Univariate		Multivariate	
	*b* (SE)	*P*-value	*b* (SE)	*P*-value
Age (years)	0.01 (0.150)	0.957		
Gender	-0.288 (0.084)	0.001*	-0.281 (0.083)	0.001*
Mean spherical equivalent (diopter)	0.269 (0.146)	0.068		
Mean axial length (mm)	0.037 (0.055)	0.500		

## Discussion

Normal reference values for ONH and RNFLT parameters were presented using Cirrus SD-OCT in normal Malay children aged seven to 17 years. Earlier studies used the time-domain OCT as a gold standard in their research [8-10]. However, the use of SD-OCT higher definitions has recently become more widespread. A comparison of ONH and RNFL measurement studies using OCT in normal children with almost similar age groups is shown in Table 5 [1-10]. To our awareness, this is the first analysis in Southeast Asian countries that highlights the normative values for ONH and RNFLT parameters, the associations between these parameters, and the factors influencing Malay children using Cirrus SD-OCT.

**Table 5:** Comparison of studies on ONH parameters and RNFLT measurements by OCT in normal children and young adults. ^*^Mean (range). ^a^Dominant eye. ^s^Superior. ^t^Temporal. ^i^Inferior. ^n^Nasal. U, undetermined; ONH, optic nerve head; RNFLT, retinal nerve fiber layer thickness; OS, oculus sinister (left eye); IT, inferior temporal; IN, inferior nasal; OCT, optical coherence tomography; OD, oculus dexter (right eye)

Author	n	Age (years)	Disc area (mm^2^)	Rim area (mm^2^)	CDR^a^	RNFLT^a^ (µm)	RNFLT^s^ (µm)	RNFLT^t^ (µm)	RNFLT^i^ (µm)	RNFLT^n^ (µm)	OCT version
This study (Malaysia, 2016)	95	10.6 (2.82)	2.32 (0.40)	1.53 (0.33)	0.55 (0.13)	102.08 (11.08)	131.05 (20.2)	72.24(11.06)	134.14 (18.54)	70.71 (12.36)	Cirrus
		(Range 7-17)									
Elia et al. [1] (Spain, 2012)	344	9.2 (1.7)	2.05 (0.39)	1.59 (0.33)	0.43 (0.19)	98.5 (10.8)	123.6 (19.5)	69.4 (11.3)	130.2 (18.1)	71.3 (13.5)	Cirrus
		(Range 6-13)									
Al-Haddad et al. [2] (Lebanon, 2013)	108	10.7 (3.1)	U	U	U	95.6 (8.7)	120.6 (13.8)	66.4 (8.9)	124.8 (18.1)	70.1 (13.0)	Cirrus
		(Range 6-17)									
Barrio-Barrio et al. [3] (Spain, 2013)	283	9.6 (3.1)	U	U	U	97.4 (9.0)	124.7	67.4	128	69.7	Cirrus
		(Range 4-17)									
Tariq et al. [4] (Australia, 2012)	1,521	17.3 (0.6)	1.98 (0.38)	1.50 (0.30)	0.44 (0.18)	99.4 (9.7)	124.8 (15.9)	69.8 (11.5)	128.5 (17.2)	74.3 (12.6)	Cirrus
		(Range 16-19)									
Turk et al. [5] (Turkey, 2012)	107	10.7 (2.9)	U	U	U	106.46 (9.41)	ST 139.0 (17.6)	74.31 (9.45)	IT 144.6 (17.2)	71.54 (10.03)	Spectralis
		(Range 6-16)					SN 116.2 (16.0)		IN 106.4 (19.1)		
Yanni et al. [6] (North America, 2013)	83	8.9*	U	U	U	107.6 (1.2)	ST 145.1 (2.2)	76.5 (1.9)	IT 147.0 (2.1)	84.5 (1.9)	Spectralis
		(Range 5-15)					SN 116.2 (2.8)		IN 125.4 (3.0)		
Tsai et al. [7] (Taiwan, 2012)	470	9.2*	U	U	U	109.4 (10.0)	133.9 (18.1)	90.4 (14.3)	142.2 9 (19.5)	71.1 (11.3)	RTVue-100
		(Range 6.5-12.5)									
Leung et al. [8] (Hong Kong, 2010)	97	9.7*	U	U	U	OD 113.5 (9.8)	OD 146.3 (16.3)	OD 87.3 (15.4)	OD 142.4 (18.4)	OD 78.3 (16.1)	Stratus
		(Range 6.1-17.6)				OS 113.1(10.8)	OS 148.6 (19.5)	OS 86.6 (16.6)	OS 143.2 (8.7)	OS 74.2 (14.8)	
Pawar et al. [9] (India, 2014)	120	10.8 (3.2)	U	U	U	106.11 (9.51)	133.44 (15.50)	70.72 (14.81)	134.1 (16.16)	84.27 (16.43)	Stratus
		(Range 5-17)									
Samarawickrama et al. [10] (Australia, 2009)	3,382	6	U	1.76 (0.42)^n^	0.20 (0.13)^a^	104.5 (11.0)^a^	U	U	U	U	Stratus
		12		1.93 (0.42)^n^	0.19 (0.14)^a^	104.1 (10.8)^a^					

We measured a mean disc area of 2.32 (0.40) mm^2^ and a mean rim area of 1.53 (0.33) mm^2^. Our findings aligned with Elia et al., who reported a mean disc area of 2.05 (0.39) mm^2^ and a mean rim area of 1.59 (0.33) mm^2^ in children in Spain. Similarly, the average mean of RNFL in our Malay children was comparable to the data from Taiwan, Turkish, Indian, and East Asian children living in Australia [5,7,9,10]. However, calculations were made using Spectralis OCT and the earlier version of the time-domain OCT. It is important to note that Tariq et al. and Samarawickrama et al., who studied a large sample of Australian children aged between six and 19 years, recorded that East Asian children had a thicker RNFL, a larger disc area, a smaller rim area, and a larger CDR than white children [4,14]. On the other hand, retinal thicknesses of less than 100 μm were found in children living in Lebanon, Spain, and Australia [1-4].

Our study showed a slightly different distribution of RNFL, with the inferior part being the thickest followed by the superior, temporal, and nasal parts being the thinnest. This finding was consistent with the 2012 reports by Turk et al. and Tsai et al., who conducted a study on Turkish and Chinese children in Taiwan with different models of OCT, Spectralis, and RTVue-100 [5,7]. A few previous reports in the United States and Australia showed an exception to the normal ISNT (Inferior, Superior, Nasal, and Temporal) rule [15-17].

This research indicates a substantial association between gender and the optical disc area (*b *= 0.281, *P *= 0.001), with males having a greater disc area relative to females. Tariq et al. observed a similar gender influence on optical disc parameters [4]. Interestingly, in 2009, Pang et al. observed substantial discrepancies between the genders in the CDR and the linear CDR over the standard ONH parameters. In this research, these two parameters were higher in boys than in girls of African American children [18]. This observation was confirmed by He et al., who studied Chinese children aged seven to 15 years, and found that the disc area and cup area were smaller in girls relative to boys [19]. No major age, axial duration, or SE effects on other ONH parameters have been observed.

We stated that there was no substantial association between gender and RNFLT. This result was comparable to previous published reports, which found that there was no significant gender influence on RNFLT [1,3,9,17]. We also did not find a correlation between age and RNFLT. This result was consistent with other published research [1,2,5,8,9,14,18]. On the other hand, Hsu et al. stated that age was associated negatively with the rim parameters, average RNFL, and superior RNFL in 133 patients aged 20 to 77 years in Taiwan. However, the reviewer concluded that the aging impact on the neuroretinal rim and RNFL was nonuniform and that age was not a consistent confounding factor by using OCT [20].

A few studies investigated the relationship between ONH and RNFLT parameters with axial length [3,5,21,22]. No major influence of axial length on ONH and RNFLT parameters was observed. This discovery was parallel to studies by Barrio-Barrio et al. and Turk et al., which published related findings [3,5]. On the other hand, in a large study involving six-year-old children living in Australia, Huyn et al. reported a substantial increase in the optical disc area and a decrease in the axial rim area [21]. In 2007, Budenz et al. reported a thinner mean RNFLT of approximately 2.2 µm for every 1 mm increase in the axial length in 328 average adult subjects in the United States [22]. These negative associations were subsequently confirmed by El-Dairi et al. in 2009 [15] and Tariq et al. in 2012 [4]. Earlier researchers used the time-domain OCTs for various age ranges, potentially leading to different results compared to ours. RNFL thinning with longer axial length can also be due to the magnification effect [4].

The effect of refraction on OCT measurements has been discussed in the literature. There was no meaningful association between SE refraction and these measurements. This was predicted because we only included emmetropic children within a range of ± 0.5 DS in our sample. This finding was also similar to research performed by Turk et al., Tong et al., and Pang et al. who studied children aged between six and 17 years, with SE varying from -8.0 to +6.0 diopters [5,11,18].

We reported an important association between the RNFLT and the disc area as well as the rim area. The inferior RNFLT was negatively correlated with the average CDR (*r *= -0.238, *P *= 0.020). This result was consistent with Tariq et al., who observed a strong association between the disc region, the rim area, and the CDR with the average RNFL [4]. Tariq et al. stated that the larger disc and rim area was associated with the thicker RNFL, while the larger CDR was associated with the thinner RNFL.

Previous studies in healthy patients using the time-domain OCT (Stratus OCT) found a strong association between RNFLT and optical disc size [23,24]. This finding may be attributed to an increase in the number of optic nerve fibers associated with the larger size of the ONH or a narrower gap between the fixed circular scan and the true ONH margin. A study by Jun et al. on Koreans, involving ages between 4 and 15 years, documented a decrease in RNFL with a smaller disc area [23]. A similar finding was observed in another study conducted by Savini et al. among older patients in Italy, with ages ranging from 15 to 54 years [24]. However, Mansoori et al. reported conflicting results in Indian adults [25].

The strength of our study lies in providing new evidence on ONH and RNFL analysis in Southeast Asian children using the new generation of Cirrus SD-OCT. The use of Cirrus SD-OCT results in less variation in repetitive measurements, demonstrating 22 greater sensitivity and accuracy compared to HRT. In comparison, this study included a wide range of age groups, spanning from seven to 17 years. All children underwent a comprehensive eye examination conducted by a single qualified researcher and optometrist. Detailed associations between all parameters have also been reported in this analysis. The main limitation of our analysis is that it is restricted to a single ethnic community.

## Conclusions

In conclusion, this study presents normative evidence for ONH and RNFLT parameters in emmetropic Malay children measured using Cirrus SD-OCT. In emmetropic Malay individuals, males exhibited a larger optical disc region compared to females. The increase in RNFLT was correlated with a significant increase in disc and rim area. These findings advocate for the measurement of ONH and RNFLT, especially in children suspected or diagnosed with glaucoma and optic neuropathy.
